# Persistence of TBT and copper in excess on leisure boat hulls around the Baltic Sea

**DOI:** 10.1007/s11356-018-1614-1

**Published:** 2018-03-12

**Authors:** Britta Eklund, Burkard Watermann

**Affiliations:** 1 Department of Environmental Science and Analytical Chemistry, ACES, Stockholm University, Stockholm, Sweden; 2 Laboratory for Aquatic Research, Limnomar, Hamburg, Germany

**Keywords:** TBT, Copper, Concentrations on boat hulls, XRF-measurements

## Abstract

A handheld XRF-analyzer specially calibrated for measurements of metals on plastic boat hulls has been used on leisure boats in Denmark (DK), Finland (FI), and Germany (DE). The results on tin and copper are presented as μg metal/cm^2^. Tin is a proxy for the occurrence of organotin compounds on the boat. Two or three sites were visited in each country and between 25 and 90 boats were measured at each site. Every boat was measured at six to eight places, and the results are presented both as mean and median values. Linear regression of mean to median values of the 377 data pairs shows high relationship with *R*^2^ = 0.9566 for tin and *R*^2^ of 0.9724 for copper and thus both ways of calculation may be used. However, for regulative use, it is suggested that all individual measurements on each boat should be presented and used for decisions of removal or sealing of boat hulls. The results are compared with published data from different parts of Sweden, i.e., boats in fresh water, brackish water, and salt water. The results show that tin with mean values > 50 μg Sn/cm^2^ is still found on 42, 24, and 23% of the boats in DK, FI, and DE, respectively. The corresponding percentages based on median values are 38, 22, and 18% for DK, FI, and DE, respectively. The variation among boats is high with a maximum mean value of 2000 μg Sn/cm^2^. As comparison, one layer of an old TBT antifouling paint Hempels Hard racing superior, corresponds to 300 μg Sn/cm^2^. The percentage of boats with tin > 400 μg Sn/cm^2^ content based on mean values was 10% in DK, 5% in FI, and 1% in DE. The corresponding median values were 9, 6, and 1% for DK, FI, and DE. Copper, > 100 μg Cu/cm^2^, was detected on all measured boats in DK and in DE and on all but 3% of the FI boats. One layer of Hempels MilleXtra corresponds to ~ 4000 μg Cu/cm^2^. The recommendation on the can is to apply two layers. The proportion of boats with higher mean copper values than 8000 μg Cu/cm^2^ was 51, 56, and 61 for boats in DK, FI, and DE, respectively. The proportion based on median values > 8000 μg Cu/cm^2^ was 50, 54, and 61% for DK, FI, and DE. The conclusion is that many leisure boats around the Baltic Sea still display or possess antifouling paints containing organotin compounds and that more than half of the boats have more copper than needed for one boat season according to the paint producers. Much of these known toxic compounds will probably be released into the environment and harm the biota. The calibrated XRF-method, intended for area measurements on boat hulls, is an easy and cheap way to detect boats with organotin compounds and high copper content. We recommend environmental authorities to use this method for identification of such boats and to use the results for requesting measures to minimize further leakage to the environment.

## Introduction

The Baltic Sea is a polluted sea with many hazardous substances at elevated levels in both water, sediment, and biota. One of the substances of high concern is the occurrence of organotin compounds where tributyltin (TBT) is the most important substance. In spite of prohibition of TBT since 1989 for use on leisure boats shorter than 25 m (Council Directive 89/677/EEC) and on ships from 2008 (Gipperth 2009), TBT is still detected in water, biota, and sediments in particular boat harbors and in shipping lanes. High content of TBT is one reason why many sediments do not reach “Good Environmental Status” according to the Marine Strategy Framework Directive (2008/56/EC) adopted by EU in 2008. The occurrence of organotin compounds is still high in sediments around the world (e.g., Hoch 2001; Viglino et al. 2004; Antizar-Ladislao 2008; Eklund et al. 2008, 2016; Cornelissen et al. 2008). Within the European Water Framework Directive, the organotin substances are prioritized and should be phased out as quickly as possible (Council Directive 2000/60/EC). Nevertheless, chemical and biological monitoring data show persistence and bioavailability of organotin compounds on a low but significant level around the Baltic Sea (Eklund et al. 2008, 2010, 2016; HELCOM 2009; Strand 2009; Nyberg et al. 2014). The highest values are found in harbors but even deep water sediments are considered having “bad status” with levels higher than the threshold value of > 50 μg/kg ww (HELCOM 2010). One important source of TBT is waste from maintenance work on boat hulls with old coatings on leisure boats (Eklund and Eklund 2014; Eklund et al. 2014). Elevated concentrations of TBT in sediments in leisure boat harbors have been observed in a number of studies such as by Maguire (2000), Hoch (2001), and Eklund et al. (2008, 2010). The concentrations were much above a predicted no-effect concentration (PNEC) value for TBT in sediment of 0.02 μg/kg dw (Anon 2005) and Swedish limit value of 1.6 μg TBT/kg dry sediment (Anon 2015a). These high concentrations in sediments cannot exclusively be explained by historical inputs but by persistent leaching out of old TBT paints that is enhanced when hulls are treated with high-pressure hosing (Eklund et al. 2008). The backbone polymers of eroding or self-polishing biocidal antifouling paints build no water barrier and hydrolyze at the water contact including penetration by water. Thus an overlaying copper paints with the same matrix property are penetrated by water and even the under laying old TBT paint layer. It can be expected that the leaching rate of TBT from an old paint layer beneath is lower than the top layer but trials with high pressure hosing show that even below copper paint essential amounts of TBT of old layer can be found in the waste water (Ytreberg 2012). Thus leaching of existing TBT paints occurs. These observations imply that the obligation to remove or to seal existing TBT paints according to the IMO AF-Convention (IMO 2008) has not been effected on leisure boats around the Baltic Sea.

Today, antifouling paints based mainly on copper, in some countries boosted by organic co-biocides, are the most commonly used coatings (Brooks and Waldock 2009). The general habit in most countries is that before launching, the hull is coated with new antifouling paint each year, and most boat owners are following the instructions on the can where often two layers are recommended to be applied by brush or roller. However, it is commonly observed that the paint thickness on the boat hulls increases each year, and that not all paint is leaking out during the boat season. This practice indicates the excess use of antifouling paints. With better control of the amount of paint applied on the boat hull each year, the performance could be just as good with less toxic compounds distributed into the sea.

Formerly, the only way to know the amount of toxic substances on the boat hull was to scrape off paint and send to a laboratory for chemical analysis. With the calibrated method developed by Ytreberg et al. (2015) intended for area measurements on plastic boat hulls, the concentration of metals on the boat hulls can be screened. In this method, a handheld X-ray fluorescence (XRF) analyzer was used for determining the quantity of metals in the antifouling paint on boat hulls. The advantage with this method is that it is calibrated to give the results in μg metals/cm^2^ instead of in percent as in other commonly used XRF methods. The results in metals per area enable comparisons between boats and within the same boat at various times. The method is a cost efficient, non-destructive, and rapid screening method, and for the first time boats with tin (TBT) on the hull can be identified. This makes it possible for environmental authorities to require measures to be taken and by this stop further leakage to the environment.

The method has until now, only been used in one study for screening of ca 700 boat hulls in Sweden (Ytreberg et al. 2016). The highest concentrations of both tin and copper were detected on boats moored in salt water compared to the boats in brackish water or fresh water. The question is whether this hold true for a larger region like the Baltic Sea and how this complies with regulation in different areas and countries. This is investigated in this study.

## Aim

The main object was to investigate the persistence of tin, on boat hulls in Finland, Denmark, and Germany and to compare the results with Swedish data. Another aim was to measure the amount of copper on boat hulls in the countries around the Baltic Sea and relate to the compliance with existing regulation in the countries. Furthermore, the readings delivered data on regional and national practice in boat maintenance.

## Methods

A handheld X-ray Fluorescence (XRF) analyzer calibrated for area measurements of tin, copper, and zinc in antifouling paints coated on plastic boat hulls (Ytreberg 2012) has been used to measure metals on leisure boat hulls in Finland (FI), Denmark (DK), and Germany (DE) in the period April 2015 to February 2017. The Olympus XRF-analyzer (Delta-50, Innov-X) is equipped with a 4 W, 50 kV X-ray tube, which is able to excite and detect heavy elements such as the K-lines of tin. The penetration depth differs depending on the element itself and the combination of other elements in the sample. With lower total metal content measurement of tin has been shown to be linear up to ca 120 μm dry film and with higher total metal content, it was linear up to 55 μm dry film (Ytreberg et al. 2017b). With thicker samples and higher total metal content, it is increasingly underestimating the concentration of metals up to a thickness maximum of 600 μm dry film (Ytreberg et al. 2017b).

Three sites were visited in Finland along the southern coast, and the number of measured leisure boats was 52, 53, and 51, respectively. The salinity was around 4–5 practical salinity unit (PSU) at all these sites. In Denmark, three boat clubs near Helsingör at the Öre sound were measured with 25, 28, and 28 boats at the respective sites. The salinity in Öre sound is in the range 12 to 15 PSU. In Germany, two boat clubs in the Kiel bay were visited, one with 50 boats and a storage hall with 90 boats from five different harbors. The salinity ranges between 12 and 20 PSU. The selection of boats was either that all boats at the visited site were measured or a random selection based on every third boat or equivalent was measured. The permission to measure the boats was provided by the harbor masters of the individual boat clubs.

Each boat hull was measured at six to eight places. The places were always measured in the order starboard back, starboard middle, starboard front, portside front, portside middle, portside back, and two measuring points on the rudder or stern. The position of the measuring points on sailboat hulls was ca 20 cm below the water line. On motor boats, the bottom of the hull was measured on starboard and portside and on the stern at the side ca 10 cm below the waterline. The measuring time at each place was 10 s where the 50 kV beam of the instrument was used for the entire period. In the calculation, both the mean and the median values of all measurements per boats have been compared.

The data has been compared to existing data from Sweden performed in 2015 at the West coast of Sweden (20 PSU), in the Stockholm area (5–6 PSU), and in freshwater (Ytreberg et al. 2016). In this study only one place per boat was measured.

The performance of the instrument was checked at least twice each day of measurement by shooting at two reference samples where one contained tin and the other copper. Four measurements were conducted on each reference sample.

The data has also been compared to measurements on one layer of antifouling paints applied by a roller on a plastic piece from a boat hull. For tin, an old but newly opened can from Hempel (Hard Racing Super, no 4182) containing bis (TBTO) and tributyltin fluoride) was used and for copper, the commonly used antifouling paint Hempel MilleXtra (71100) containing 34% copper-containing (di copper oxide) paint) was used.

## Results

**Fig. 1:**
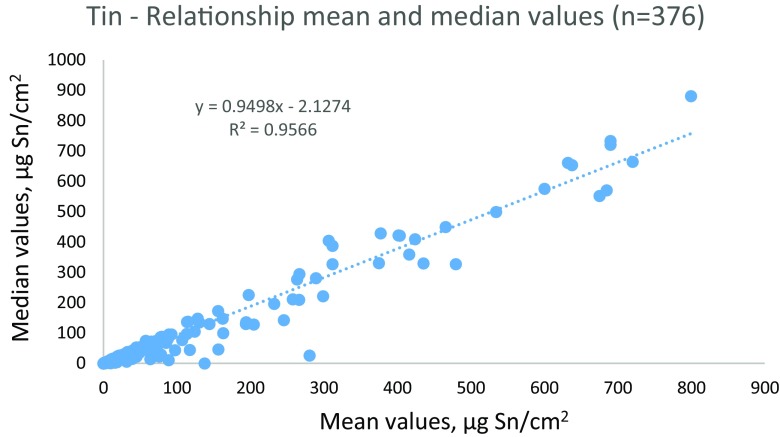
Relationship between mean and median values of tin from 376 measured boat hulls (6–8 measurements per boat hull) in Denmark, Finland, and Germany. (One very high value with mean and median around 2000 was excluded in the graph)

**Fig. 2:**
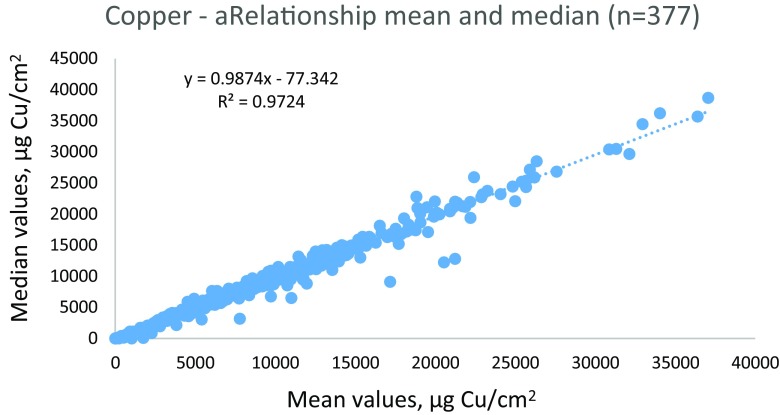
Relationship between mean and median values of copper from 377 measured boat hulls (6–8 measurements per boat hull) in Denmark, Finland, and Germany

At all investigated sites in all three countries, the majority of boats were coated with biocide containing antifouling paints. In all, 377 boats were investigated. The relationship between the mean and the median values for tin and copper mesurements is illustrated in Figs 1 and 2. The results show a linear relationship with *R*^2^ for tin 0.9735 but when one boat with very high values of around 2000 was excluded, the *R*^2^ was 0.9566 (Fig. 1). For copper, the relationship was even better with a *R*^2^ of 0.9724 (Fig. 2).

**Fig. 3:**
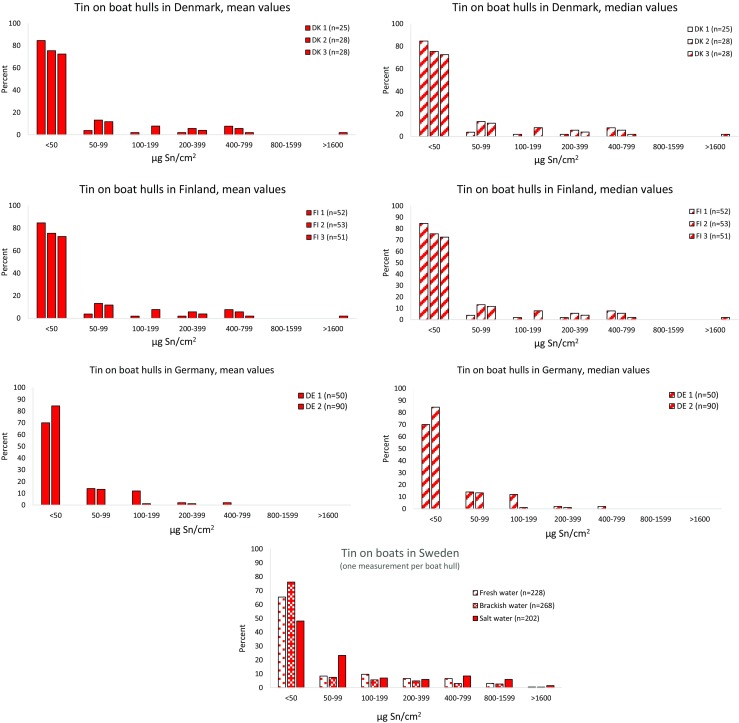
Proportion of tin on boat hulls in Denmark, Finland, and Germany (7–8 measurements per boat hull) in comparison to Swedish data (one measurement per boat hull) from Ytreberg et al. 2016

**Fig. 4:**
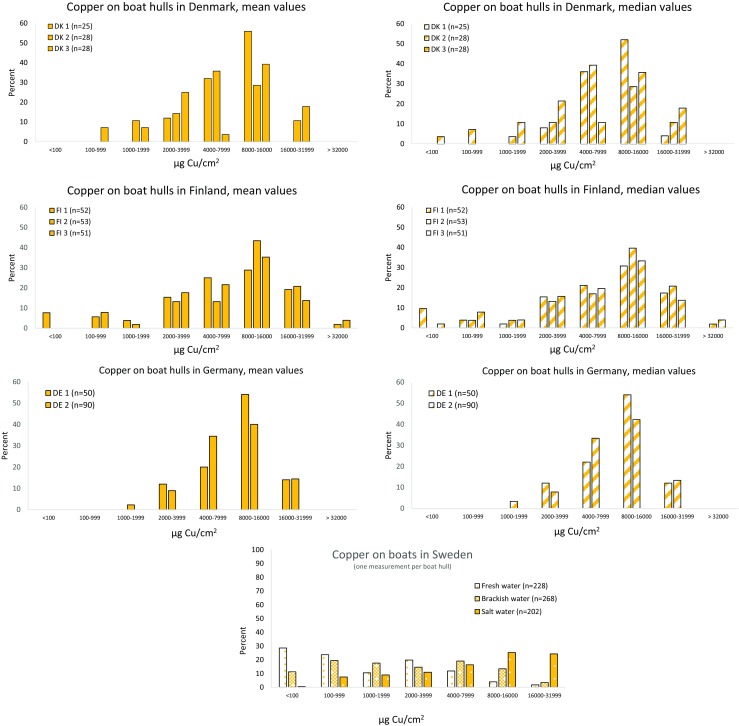
Proportion of copper on boat hulls in Denmark, Finland, and Germany (6–8 measurements per boat hull) in comparison to Swedish data (one measurement per boat hull) from Ytreberg et al. 2016. Both mean and median values are shown

The mean and median values of 6–8 measurements per boat for tin and copper are shown in Figs. 3 and 4, and a summary of the data are presented in Table 1. All boats where tin was detected, also contained copper.

**Table 1:** Percentage of boats with mean and median values (6–8 measurements) of tin on plastic boat hulls

Country and No. of sites	Mean values of tin				Median values of tin			
	< 50 μg Sn/cm^2^ St dev in brackets	> 100 μg Sn/cm^2^ St dev in brackets	> 400 μg Sn/cm^2^ St dev in brackets	> 800 μg Sn/cm^2^ St dev in brackets	< 50 μg Sn/cm^2^ St dev in brackets	> 100 μg Sn/cm^2^ St dev in brackets	> 400 μg Sn/cm^2^ St dev in brackets	> 800 μg Sn/cm^2^ St dev in brackets
Denmark 1 (*n* = 25)	56	24	8	0	64	16	8	0
Denmark 2 (*n* = 28)	57	21	11	0	61	21	7	0
Denmark 3 (*n* = 28)	61	29	11	3.6	61	25	11	3.6
Mean DK (*n =* 3)	*58* (*2.5*)	*25* (*4*)	*10* (*1.6*)	*1.2* (*2.1*)	*62* (*2*)	*21* (*4.5*)	*8.7* (*2.1*)	*1.2* (*2.1*)
Finland 1 (*n* = 52)	81	13	8	0	85	12	8	0
Finland 2 (*n* = 53)	74	11	4	0	76	11	6	0
Finland 3 (*n* = 51)	73	16	4	2	73	14	4	2
Mean FI (*n =* 3)	*76* (*4.5*)	*13* (*2.5*)	*5* (*2.3*)	*0.7* (*1.2*)	*78* (*6.2*)	*12* (*1.5*)	*6* (*2*)	*0.7* (*1.2*)
Germany 1 (*n* = 50)	70	16	2	0	74	12	2	0
Germany 2 (*n* = 90)	84	2	0	0	89	1	0	0
Mean DE (*n =* 2)	*77* (*10*)	*9* (*9.9*)	*1* (*1.4*)	*0* (*0*)	*82* (*11*)	*6.5* (*7.8*)	*1* (*1.4*)	*0* (*0*)
SE, freshwater (*n* = 202)	65	26	10	4	65	26	10	4
SE, brackish water (*n* = 268)	76	16	6	3	76	16	6	3
SE, marine water (n = 202)	48	29	16	7	48	29	16	7
Mean Sweden (*n =* 3)	*63* (*14*)	*24* (*6.5*)	*11* (*5.0*)	*5* (*2.4*)	*63* (*14*)	*24* (*6.5*)	*11* (*5.0*)	*5* (*2.4*)

Data for Sweden are from Ytreberg et al. 2016 and based on a single measurement per boat

LOQ = 50 μg Sn/cm^2^

The mean values are presented in italic with the standard deviation in brackets

The results of the quality procedure were for the means of all means of the tin sample 599; the means of all mean standard deviations was 9 and the coefficient of variations was 1.4. For copper, the mean of all means was 4199; the mean of the standard deviations was 65 and the coefficient of variation was 1.6.

The results of one layer of antifouling paints on plastic pieces from the tin paint Hard Racing Super gave a measured value of approximately 300 μg Sn/cm^2^, and the measurement of the copper antifouling paint MilleXtra gave a value of approximately 4000 μg Cu/cm^2^.

## Tin

The limit of quantification (LOQ) for field measured tin was estimated to 50 μg Sn/cm^2^. The majority of boats in all four countries had tin below 50 μg Sn/cm^2^, i.e., 58, 76, and 77% as mean value and 62, 78, and 82% as median values for all measured sites in DK, FI, and DE, respectively (Fig. 3 and Table 1). Mean values higher than 100 μg Sn/cm^2^ were found on 25, 13, and 9% of the boats, and higher than 400 μg Sn/cm^2^ were found on 10, 5, and 1% of the boats in DK, FI, and DE, respectively. The corresponding percent of median values higher than 100 μg Sn/cm^2^ was 21, 12, and 6.5, and higher than 400 μg Sn/cm^2^ was 8.7, 6, and 1 for boats in DK, FI, and DE, respectively. In Sweden, the mean value for all measured boats with less than 50 μg Sn/cm^2^ was 63%, and the respective percentage for fresh water, brackish water, and salt water was 65, 76, and 48. The Swedish values for boats higher than 100 μg Sn/cm^2^ were 26, 16, and 29 for fresh water, brackish water, and saltwater, and for boats with higher values than 400 μg Sn/cm^2^, the corresponding figures were 10, 6, and 16% (Ytreberg et al. 2016). The variation among boats is high, and a maximum mean value of 2000 μg Sn/cm^2^ was found on a Finnish boat, which corresponded to the maximum median of 1900 μg Sn/cm^2^. The maximum means at the other Finnish sites were 640 and 680 μg Sn/cm^2^, and the maximum medians were 650 and 550 μg Sn/cm^2^. In Denmark, the maximum values were 690, 690, and 800 μg Sn/cm^2^ at the respective three sites, and the median values were respectively 720, 730, and 880 μg Sn/cm^2^. The lowest maximum values were found in Germany with 530 μg Sn/cm^2^ at one site and 260 μg Sn/cm^2^at the other site. The German corresponding median values were 500 and 280 μg Sn/cm^2^. The highest maximum values were found in Sweden with 3000 μg Sn/cm^2^ on one boat at the West coast, 1700 μg Sn/cm^2^ on a boat sailing in Brackish water, and 2100 μg Sn/cm^2^ on boat at a storage area near the lake Mälaren.

## Copper

The limit of quantification (LOQ) for field measured copper was estimated to 100 μg Cu/cm^2^. The majority of boats had a mean copper value higher than 100 μg Cu/cm^2^ with 100% of the boats in Denmark and Germany and 97% in Finland (Fig. 4 and Table 2). When based on median values, the percentage boats with < 100 μg Cu/cm^2^ was 99, 96, and 100 in DK, FI, and DE. The proportion of boats with higher mean copper values than 8000 μg/cm^2^ was 51, 56, and 61% for boats in DK, FI, and DE, respectively, and the corresponding median values were 50, 54, and 61% for DK, FI, and DE (Table 2). In Sweden, the percentage boats with less than 100 μg Cu/cm^2^ were 29, 11, and 1% for fresh water, brackish water, and salt water, respectively. The Swedish proportion of boats with higher values than 8000 μg Cu/cm^2^ were 6, 17, and 55% for fresh water, brackish water, and saltwater. Also for copper, the variation among boats is large. The maximum mean value of 37,100 μg Cu/cm^2^ was found at one of the Finnish boat clubs, and the maximum median value was 38,700 μg Cu/cm^2^. The mean maximum copper values at the other two sites were 24,800 and 34,100 μg Cu/cm^2^, and the median maximum were 25,900 and 36,200 μg Cu/cm^2^. The German boat sites had similar maximum mean values of 32,100 and 33,000 μg Cu/cm^2^ with median maximum of 29,700 and 34,500 μg Cu/cm^2^. The highest mean value in Denmark was 26,300 μg Cu/cm^2^, and the maximum at the other sites were 16,000 and 19,500 μg Cu/cm^2^, respectively. Based on the median values, the corresponding maximum for the Danish boats were 28,500, 16,200, and 21,100. The Swedish maximum values were again at the top with 58,000 μg Cu/cm^2^ for a boat at the West coast, 46,200 μg Cu/cm^2^ at the brackish water site, and 25,500 μg Cu/cm^2^ for boats in the lake Mälaren.

**Table 2:** Percentage of boats with mean and median values (6–8 measurements) of copper on plastic boat hulls

Country and no. of sites	Mean values of copper % boats in different ranges			Median values of copper % boats in different ranges		
	< 100 μg Cu/cm^2^ St dev in brackets	> 4000 μg Cu/cm^2^ St dev in brackets	> 8000 μg Cu/cm^2^ St dev in brackets	< 100 μg Cu/cm^2^ St dev in brackets	> 4000 μg Cu/cm^2^ St dev in brackets	> 8000 μg Cu/cm^2^ St dev in brackets
Denmark 1 (*n* = 25)	0	88	56	0	92	56
Denmark 2 (*n* = 28)	0	75	39	0	79	39
Denmark 3 (*n* = 28)	0	61	57	4	64	54
Mean DK (*n = 3*)	*0*	*75* (*14*)	*51* (*10*)	*1.3* (*2.3*)	*78* (*14*)	*50* (*9.3*)
Finland 1 (*n* = 52)	8	73	48	10	69	48
Finland 2 (*n* = 53)	0	79	66	0	79	62
Finland 3 (*n* = 51)	0	75	53	2	71	51
Mean FI (*n = 3*)	*3* (*4.6*)	*76* (*3.2*)	*56* (*9.3*)	*4* (*5.3*)	*73* (*5.4*)	*54* (*7.5*)
Germany 1 (*n* = 50)	0	88	68	0	88	66
Germany 2 (*n* = 90)	0	89	54	0	89	56
Mean DE (*n* = 2)	*0*	*88* (*0.6*)	*61* (*9.6*)	*0*	*88* (*0.6*)	*61* (*7.4*)
SE, freshwater (*n* = 202)	29	18	6	29	18	6
SE, brackish water (*n* = 268)	11	37	17	11	37	17
SE, marine water (*n* = 202)	1	72	55	1	72	55
Mean Sweden (*n* = 3)	*14* (*14*)	*42* (*27*)	*26* (*26*)	*14* (*14*)	*42* (*27*)	*26* (*26*)

Data for Sweden are from Ytreberg et al. 2016 and based on a single measurement per boat

LOQ = 100 μg Cu/cm^2^

The mean values are presented in italic with the standard deviation in brackets

The content of copper differs among the countries and the areas. As a summary, the highest median concentrations of copper were found on boats at the West coast of Sweden (9500 μg/cm^2^) > Helsingör at the Sound (8000 μg/cm^2^) > Helsinki in the Finnish Bay (7500 μg/cm^2^) > Stockholm area (2400 μg/cm^2^) and lowest in freshwater (900 μg/cm^2^).

## Discussion

The XRF-method, calibrated for area measurements of metals on boat hulls (Ytreberg et al. 2016), used in this study has been shown to effectively identify those boats with antifouling paints containing tin and copper. The validity of the assumption that tin corresponds to the amount of organotin compounds has been shown by Lagerström et al. (2017) who found a linear relationship (*R*^2^ = 0.934) between the total content of tin and the sum of chemically detected organotin compounds in layers of antifouling paints scraped from painted hulls of leisure boats. Therefore, higher tin values are considered to pose a risk for leakage of organotin compounds to the environment.

In this study, eight measurements per boat hull have been measured, and both the mean and the median values have been used to compare the results from the different countries. The high relationships for both tin and copper show that both mean and median values may be used for comparison between the different countries and different sites. It also indicates that usually the measured contents on the boat hull are rather even. The reason for the few discrepancies is the deviation of one or two data on the boat. Usually, this is often the case for the rudder/stern, which may differ from the rest of the boat hull, either much higher metal content or much lower. This is important information to the boat owner, and it is therefore suggested that when the XRF method is used for regulative screening of boats, all individual data should be reported and not only the mean or median value.

The penetration depth of the signal depends on the thickness of the paint layer and on the composition of other metals (Ytreberg et al. 2017b). For these reasons, the method may underestimate the amount of metals at thicker samples and in particular at high concentrations of several metals. Therefore, the calibrated XRF-method has to be regarded as a screening tool. An advantage of the method is the low cost, where a total of eight measurements of organotin compounds and copper on one boat is much less than the cost of just one sample of the corresponding chemical analysis. The calibrated XRF-method is easy to use and could be a useful tool in regulative work by environmental authorities.

In accordance with the results from Sweden (Ytreberg et al. 2016), there are no indications of freshly applied TBT-paints by illegal sale and application. Most likely the measured tin concentrations on the leisure boat hulls indicate the persistence of old TBT-paints probably as remnants in under laying paint layers after incomplete removal (Lagerström et al. 2017). Even if many boat clubs have rules for protection of the ground, most boat clubs do not enforce this regulation. In a compilation of results from 34 investigated boat storage areas in Sweden, organotin compounds have been shown to be spread and accumulated to the unprotected ground along with scraping and sanding during maintenance of the boat hulls (Eklund and Eklund 2014). The risk is evident that these biocides from antifouling paints on the boats may be spread to adjacent waters in connection with rain and run-offs, which is illustrated by results in soil and sediment at a boatyard studied by Lagerström et al. (2016).

## TBT

The differences among the four countries were large when it comes to the proportion of boats with higher tin values. One layer of TBT paint is around 300 μg/cm^2^. Mean values of tin > 400 μg/cm^2^ was found on 10% of the Danish boats and in 16% of the Swedish boats measured at the West coast, whereas in Finland, only 5% and in Germany, only 1% of the boats had higher tin content than 400 μg/cm^2^. The median values were almost the same (Table 1). Apparently, the enforcement of the TBT regulation has differed in the investigated countries.

Even if the overall results show that the majority of the measured boats in all four countries had concentrations below the LOQ of 50 μg Sn/cm^2^, still a high proportion of the boats have remnants on their boats with > 50 μg Sn/cm^2^, i.e., on 42, 24, 23, and 37%, in DK, FI, DE, and SE, respectively (Table 1). It was not possible to get the building year of the boats but most likely, the tin and organotin compounds are found in under-lying paint layers on boats built before the prohibition of organotin compounds in antifouling paints (Council Directive 89/677/EEC). However, it cannot be excluded that a few newer boats may have been imported from countries outside Europe with less restriction of TBT paint.

Overall, the pattern of tin occurrence on boat hulls in all four countries was alike (Fig. 1). Even in Sweden, there were no large difference between the different areas, and in fact, the highest measured tin concentration of 3000 μg Sn/cm^2^ was on a boat in fresh water. The little difference between the countries may be due to that almost all boats built before 1989, when the prohibition of TBT was enforced in EU (Council Directive 89/677/EEC), were coated with TBT paints and thus the proportion of older boats with such paints is similar.

High inputs derived from high pressure washing (hp) after lifting the boats in autumn has been determined. In a study 2007 of sediments in a gradient towards the slipway in a small harbor for recreational boats, the concentration of organotin compounds increased the closer to the uptake area (Eklund et al. 2008). In a Finnish study performed in 2015 and 2016 of wash water from a wash down area, both TBT and triphenyltin (TPhT) were detected. The values of TBT were 0.023 and 0.75 μg/L and for TPhT 0.085 and 1.4 μg/L (Haaksi and Gustafsson 2016). Measurements in wash pads in Sweden of wash water and precipitated waste from several wash pads show high amounts of biocides in the sediment including TBT with median values of 69,000 μg TBT/kg dry sediment at the West coast and 11,000 μg TBT/kg dry sediment from wash pads at the East coast (Ytreberg 2012, Ytreberg et al. 2015). These data show that TBT is still discharged in connection with washing of the hull. High-pressure washing is common practice in all four countries albeit antifouling paints except hard coatings are designed to erode or polish and cannot stand hp-washing without damage or removal of upper paint layers.

The national laws around the Baltic Sea are quite differing. In Germany, according to the Federal Water Act, it is illegal to clean boat hulls with tap or high-pressure water outside wash-down areas or tarps as collection systems and is only allowed on wash-down areas with collection and filter systems. In a few harbors at the German Baltic coast, hp-washing is prohibited. In Sweden, there is a national recommendation not to clean boats outside areas with filtering and collection systems (Anon 2015a, b). The national authority leaves it to the municipality to make the final decision about the regulation in their respective community. A similar approach is found in Denmark where the local harbors can decide on regulations for the boats in their harbor (personal communication H. Anker, Professor at Copenhagen University). Many municipalities in Finland have enforced local regulations for boat washing refereeing to the national environmental law stating that the ground and water should not be polluted (personal communication with Tomas Kull, Environmental inspector in Pargas City). In practice, it does not exist specific regulations for most of the small boat harbors in the investigated countries.

However, even if there is a global ban for use of TBT containing antifouling products, such products are still on sale in some areas, and the contamination is not decreasing (Bargar et al. 2013; Turner and Glegg 2014, https://www.seahawkpaints.com/antifouling-bottom-paint/visited at 22-08-2017).

A boat hull with an average 100 or 400 μg TBT/cm^2^ means on a hull of 10 m^2^ a total amount of 10 or 40 g. In view that as low concentrations as 1 ng/L are sufficient to affect the endocrine system of molluscs (Bryan et al. 1986), and low concentrations of TBT are harmful to other organisms (references in review by Antizar-Ladislao 2008), the amount of organotin compounds that potentially could be distributed to the environment from boat hulls is considerable.

On the background of persisting concentrations of tin on boat hulls, we suggest the following options to be enforced by environmental authorities, harbor operators, boat clubs, etc.:

Certificate for boats built before 2008 on the existing paint layers. In case of existing paints with illegal compounds like organotin, diuron, or irgarol, these boats should be strictly excluded from hp-washing outside wash-down areas. Cleaning with a sponge should be performed with a tarp to protect the ground and collection of the wash water.

Or obligation to remove all paint layers down to the primer in case no certificate can be delivered

Prohibition of hp-washing for boats from other harbors, resp. guest boats unless presenting a certificate.

In harbors where the erection of a wash-down area with collection and filter systems is too expensive, as maintenance practice should be recommended to clean the hulls after lifting in autumn with a soft sponge and sand them softly, if necessary, before launching in the next spring. These maintenance works should include protection measurements of the ground.

## Copper

The results show similar pattern of range intervals of copper in Denmark, Finland, Germany, and the Swedish West coast with most boats in the interval 8000 to 16,000 μg Cu/cm^2^, and between 50 and 61% of these boats had more than 8000 μg Cu/cm^2^ both calculated as means or as median values (Fig. 4, Table 2). This can be related to the fact that one layer of the commonly used copper-based paint Hempel MilleXtra. corresponds to ca 4000 Cu/cm^2^. Thus, two paint layers, which are recommended by the paint producers to last for a boat season, is roughly 8000 μg Cu/cm^2^. This means that most boats have more paint layers on their boats, which must be regarded as an excess of what is needed. The potential amount of copper that may leak into the environment from a single boat is thus: a boat with a hull area of 20 m^2^ and a copper concentration of 8000 μg Cu/cm^2^ will result in 1.6 kg copper and 3.2 kg copper with a concentration of 16,000 μg Cu/cm^2^. The leakage rate varies depending on the formula in the paint but also on the salinity of the water. In more saline waters, the leakage rate is faster than in less salty waters and in freshwater (Ytreberg et al. 2017a; Lagerström et al. 2018). The conclusion is still that much copper each year may leak into the environment from leisure boats. Much of the copper may be taken up by fouling organisms attached to the hull and may reach concentrations up to 28,000 mg copper/kg dw (Bighiu et al. 2017). When this material in connection with scraping and sandpapering enters the ground, it exceeds the guidance values for least sensitive land use of 200 mg copper/kg dw in Sweden by factors up to 140 and thus adds to the contamination of the soil.

In Sweden, the pattern is different depending if the boat is moored in salt water at the West coast, in brackish water in the Stockholm area, or in freshwater where biocidal antifouling paints are not allowed (Ytreberg et al. 2016). The boats with the higher copper values are found on the saltwater boats and the lowest in freshwater (Table 2). The salinity along the Finnish coast is comparable to the Stockholm water area and the fouling pressure is similar. However, the use of copper in Finland is as high as in the sites with saltwater, i.e., in Germany, Denmark, and Swedish West coast. This difference between Sweden and Finland is probably due to the different regulations in the two countries. In large, Finnish boat owners are allowed to use the same antifouling paints as in Germany, Denmark, and the West coast of Sweden, and this is apparently what they do. In Sweden, a differentiated regulation was enforced already in 1993 (Chemical Agency in Sweden 1993). Based on a risk benefit/analysis, the decision was taken that no biocide-containing antifouling paints were approved for use in freshwater and the Bothnian Bay, lower copper content was approved for the Swedish brackish water, and higher copper content was approved for use in marine waters (West coast of Sweden). The Swedish data show that the lower salinity, the higher proportion of boats with less copper. This finding supports that the regulations have had a restrictive effect. However, even if copper in antifouling paints has been prohibited for use on boats in freshwater for more than 20 years in Sweden, copper > 100 μg Cu/cm^2^ was still measured on 71% of the surveyed boats along the lake Mälaren (Ytreberg et al. 2016). One explanation is that boaters belonging to boat clubs in Lake Mälaren used their boats both in the lake and in the brackish environment and interpreted the regulation that they then were allowed to use copper paints. However, the writing of the regulation has the last year become stricter, and today it is prohibited to use antifouling paint based on copper in fresh water. The XRF-method may be a good tool for the environmental authorities to control this regulation.

The high concentrations of copper on leisure boat hulls in Kiel may be explained by several factors. Due to the high fouling pressure with barnacles and blue mussels along the German Baltic coast, German boat owners repaint the hull every year irrespective of a fail in performance of the antifouling paint in the previous season. Paint manufacturers recommend paints with copper contents of 20–25%. It is uncommon to repaint exclusively those areas of the hull which have been fouled in the previous season, and it is recommended but uncommon, to apply each paint layer in a different colors to control the polishing or erosion rate.

The new application of a handheld X-ray analyzer facilitates identification of boats with high contents of toxic heavy metals on plastic boat hulls. Work on calibrations for hulls made by steel, aluminum, and wood is on its way. The calibration in metals/cm^2^ gives figures, which can be compared with other measurements in space and time in contrast to ordinary XRF-analyzers which only provides figures in percent (Ytreberg 2012). In particular boats with high tin concentrations, indicating organotin compounds, can then be recommended for removal or sealing of the old paint in accordance with the Water Framework Directive in the EU (2000/60/EC). In addition, violation of the ban of copper-based paints in freshwater areas can be detected and identified. This would be a fast way to eliminate further discharges of these substances to the environment. Under this aspect, the XRF method can be regarded as a powerful tool for the survey and monitoring of antifouling practice.

According to the implementation of the EU-BPR and the transformation into national law, it can be expected that several copper compounds will be incorporated in authorized antifouling products of the future. Despite initiatives to reduce the copper content of antifouling paints and to phase-out biocidal antifouling paints by 2030, there will be a need for monitoring, especially in freshwater areas and the Bothnian Bay where actually no biocidal paints are allowed at the Swedish side.

## Conclusions

In comparison to chemical analyses, the XRF-method to identify tin and copper on boat hulls is a non-destructible, cheap and fast method. The calibrated screening method is recommended to be used in regulative works to provide data on which regulatory authorities can rely in determining which boats need to remove tin paint or have excess of copper paint. The need is illustrated by the fact that 23–42% of the in total 377 measured boats in Denmark, Finland, and Germany still have amount of tin (organotin compounds) on their hulls. This is a source to leakage to the environment and is one cause to elevate organotin concentrations in the sediments along the Baltic shores and a reason why good status according to the Marine Strategy Framework Directive (2008/56/EC) is not achieved.

The data from all countries show that an excess of antifouling copper is used in all countries. Approximately 50% of the measured boats in DK, DE, and FI, and at the West Coast of SE had higher concentrations on their boat hulls than two layers of the most common copper-containing antifouling paint with 34% copper. This excess of copper use should be stopped.
